# The Effectiveness of Sensory Adaptive Dental Environments to Reduce Corresponding Negative Behaviours and Psychophysiology Responses in Children and Young People with Intellectual and Developmental Disabilities: A Protocol of a Systematic Review and Meta-Analysis

**DOI:** 10.3390/ijerph192113758

**Published:** 2022-10-22

**Authors:** Kaitlyn Reynolds, Navira Chandio, Ritesh Chimoriya, Amit Arora

**Affiliations:** 1 School of Health Sciences, Western Sydney University, Campbelltown Campus, Locked Bag 1797, Penrith, NSW 2751, Australia; 2 Health Equity Laboratory, Campbelltown, NSW 2560, Australia; 3 Translational Health Research Institute, Western Sydney University, Locked Bag 1797, Penrith, NSW 2751, Australia; 4 Philanthropy Nepal (Paropakari Nepal) Research Collaboration, Auburn, NSW 2144, Australia; 5 School of Medicine, Western Sydney University, Campbelltown, NSW 2560, Australia; 6 Discipline of Child and Adolescent Health, Sydney Medical School, Faculty of Medicine and Health, The University of Sydney, Westmead, NSW 2145, Australia; 7 Oral Health Services, Sydney Local Health District and Sydney Dental Hospital, NSW Health, Surry Hills, NSW 2010, Australia

**Keywords:** dental anxiety, intellectual developmental disorder, multisensory, oral health, sensory processing

## Abstract

People with Intellectual and Developmental Disabilities (IDDs) are disproportionately vulnerable to poorer oral health due to their complex needs specifically sensory processing difficulties. This leads to increased maladaptive behaviours and psychophysiology responses of dental anxiety amplified by the overstimulating aspects of the dental environment. Although, there is a growing body of evidence to suggest that sensory adaptions are an effective strategy for individuals with IDDs in a wide range of settings, there is a lack of high-quality evidence detailing the effectiveness in a dental setting. The objective of this review is to assess the effectiveness of sensory adaptive dental environments (SADE) to reduce dental anxiety, corresponding negative behaviours and psychophysiology responses in children and young people with IDDs. The systematic review will include all Randomized Controlled Trials (RCTs) that investigate the effectiveness of SADE compared to control (no intervention), waitlist or usual care (regular dental environment) to reduce dental anxiety and the corresponding negative behaviours and psychophysiology responses in children and young people (upto the ages of 24 years) with IDDs. This review will be conducted according to the Preferred Reporting Items for Systematic Reviews and Meta-Analyses (PRISMA) guidelines. Databases including MEDLINE (Ovid), The Cochrane Library, Embase, Google Scholar, Web of Science and OT Seeker will be searched using appropriate keywords. Additionally, citation searching will be conducted. Screening based on titles and abstracts will be done after de-duplication, followed by full-text reading for selection based on the inclusion criteria. Data extracted from the included studies will be tabulated and assessed for risk of bias. If applicable, a meta-analysis of the pooled data will be conducted. The review is registered with PROSPERO (CRD42022322083).

## 1. Introduction

Intellectual and Developmental Disabilities (IDDs) are a group of conditions due to physical, learning, language, or behaviour impairments. These conditions impact day-to-day functioning and include attention deficit hyperactivity disorder, autism spectrum disorder, cerebral palsy, down syndrome, intellectual disability, learning disability, and other developmental delays as classified by the American Psychiatric Association [1]. IDDs contribute significantly to total disease burden globally [2]. A high prevalence of IDDs has been documented in the United States in a systematic review by Anderson et al. [3], with a prevalence of 69.9 per 1000 for children and 41.0 per 1000 for adults. This picture is not unique to the United States with several other studies having documented similar high prevalence in countries such as Australia [4] and the United Kingdom [5].

Evidence suggests the existence of health inequalities between people with and without IDD are closely tied to individual, environmental, social, and/or economic determinants [6]. Of particular concern is oral health for people with IDD, with research showing that this population has poorer oral health than those without, specifically significant higher levels of untreated dental caries, periodontal disease, dental plaque, worse gingival status, and fewer decayed and filled permanent and primary teeth [7,8]. The implications of poor oral health are substantial, with emerging research highlighting the destructive impact on one’s general health [9]. This vulnerability to poorer oral health in IDD population has been linked to their complex needs increasing their risk of dental disease and challenges in accessing both routine and preventative dental services [10,11].

Several studies emphasise barriers for individuals with IDDs in accessing dental care reported by individuals, families, and dental practitioners. Barriers that studies have identified include over-stimulating physical environments, challenges with waiting room, sensory processing issues, hyper-empathy, oral aversion, negative adaptive behaviours, and decreased health provider knowledge or education [10,11]. A study presented that dentists’ attitudes serve as a barrier to providing care for people with IDD including difficulties with behaviour management, inadequate training, previous experiences and severity of patient’s condition [12].

A large amount of evidence supports that dental anxiety is exacerbated by the dental environment [13,14]. Dental anxiety is a psychophysiological state described as an abnormal fear or worry in apprehension of dental treatment [15]. Dental anxiety has been described as multidimensional including somatic, cognitive, and emotional aspects [16]. Dental anxiety causes altered behaviour characterised by increased aggression and avoidance, plus physical symptoms detailed as sweating, decreased gastrointestinal motility and cutaneous vasoconstriction [17]. Dental anxiety is significant for people with IDD [15]. For IDD population, this has been linked to low compliance and psychological, cognitive, and behavioural consequences during treatment impacting overall oral health [18]. There is a growing body of evidence linking experiences of dental anxiety for children and young people with IDD to sensory processing issues. Current research has identified high prevalence of co-morbid sensory processing difficulties with modulation or discrimination issues with maladaptive behaviours for this population [19]. Sensory integration is a neurological function that processes and organises sensory modality inputs from one’s own body and the environment for functional outputs that enables an individual to engage in activities of daily living effectively [20]. Evidence emphasises the impact of sensory processing challenges on behaviour [21] which has been linked with non-compliance and reduced engagement in dental appointments and oral health practices [22]. Lee and Chang [14] uncovered that children with IDDs were particularly sensitive to the noise, smell, and visual images of sharp instruments, including needles in environment that increased their dental anxiety and refusals which is provoked by the increased sensory processing issues relating to the dental environment.

Multiple approaches are used to address these barriers such as pharmacological sedation, as well as non-pharmacological approaches including restrictive restraint boards, desensitisation, behavioural and cognitive training, video-modelling, reinforcement, distraction/relaxation, tell-show-do techniques and social stories [23]. Restrictive and pharmacological practices do not address the underlying factors that cause behaviour [6,24] and limit personal freedom, quality of life and participation [25]. Importantly, evidence highlights sedation fails to promote preventive oral care [26]. This suggests that additional measures need to be employed to increase participation in regular dental treatment to reduce oral health burden. Specifically, SADE have been studied to reduce maladaptive behaviours for the IDD population [27,28]. This approach uses a “Snoezelen room”, a well-equipped multi-sensory environment combining good lighting, mesmerising sound, deep pressure, vibration, aroma, and tactile sensation [29]. The implementation of these sensory adaptions aims to regulate sensory ‘flight or fight’ responses to reduce associated maladaptive behaviours and in-turn reduce dental anxiety [27,28].

Although, there is a growing body of evidence to suggest that SADE are effective to regulate maladaptive behaviours associated with dental anxiety, there is a lack of high-quality synthesised evidence. To inform best practice, rigorous research needs to be conducted on SADE to inform dental professionals to better address individuals with IDDs needs to reduce dental anxiety and improve oral health. Multiple scoping searches were conducted to assess previous systematic reviews in this topic area and the assessment of three reviews identified various knowledge gaps. Two reviews focused broadly on non-pharmaceutical strategies in general and therefore, only had limited information on sensory adaptive environments as an approach [23,30]. Another recent systematic review conducted by Ismail et al. [31] had poorly reported methods to replicate and population was not specific in diagnosis and the study population was without a specific diagnosis focusing generally on children 6–12 years of age. Therefore, there is no synthesis of literature that encompasses children to young people. To inform best practice, rigorous research needs to be conducted to synthesise evidence for SADE used by dentists to assist with management of children and young people with IDD. Therefore, the purpose of this systematic review is to assess the effectiveness of SADE to reduce dental anxiety and corresponding negative behaviours and psychophysiology responses in children and young people with IDD.

## 2. Review Question(s)

What is the role of SADE in providing dental treatment for children and young people (upto the ages of 24 years) with IDD?

This review aims to address the following:

- What are common sensory environmental strategies used to decrease negative behaviours and psychophysiology responses of dental anxiety in children and young people with IDD?
- Are SADE effective to reduce dental anxiety, corresponding negative behaviours and psychophysiology responses in children and young people with IDD?
- Do SADE increase children and young people with IDD participation in oral health procedures?

## 3. Inclusion Criteria

### 3.1. Participants

This review will consider studies that included participants who are children or young people up to the ages of 24 years with a diagnosis of IDD confirmed by the physicians. There was no restriction on type or severity of the IDDs.

### 3.2. Intervention(s)

This review will consider studies that evaluate sensory adaptive environments in a dental setting during oral procedures or waiting room. The interventions must aim to modulate sensory sensitivities that targets any of the senses; sight, sound, touch, smell, taste, vestibular (sense of head movement in space), proprioception (sensations from muscles and joints) and interception (sensations in relation to physiological/physical condition of the body). These strategies can include but are not limited to partially dimmed room with lighting effects, vibroacoustic, somatosensory stimuli, visual distraction, or deep pressure. Intervention can involve single or multi-sensory approaches. Dental procedures conducted in studies that involve sedation will be excluded from the review.

### 3.3. Comparator(s)

This review will consider studies that compare the intervention to control (no intervention), waitlist or usual care (regular dental environment).

### 3.4. Outcomes

This review will consider studies that measure either objective or subjective measures of cooperation, behaviour, and psychophysiology (anxiety). Primary outcomes will be categorised according to the International Classification of Functioning (ICF) [32] and adapted from the oral health framework by Faulks and colleagues [33]. These include participation restriction and body structure and function.

Participation restriction and activity participation outcome will focus on behaviour and cooperation during the dental procedure. Examples of acceptable outcome measure include compliance, cooperation, or participation scores (Frankl score, negative behaviour checklist, children’s dental behaviour rating scale or anxiety and cooperation scale), interviews or questionnaires of dentists, participants, or carers.

Body structure and function outcomes include anxiety and psychophysiology responses. Examples of acceptable outcome measure include oxygen saturation, electrodermal activity, heart rate, and skin conductance.

### 3.5. Types of Studies

This review will consider only RCTs including parallel-group, crossover, cluster, and factorial design. Non-experimental observational and non-randomised study designs including pre-poststudy designs will be excluded from this review.

## 4. Methods

The proposed systematic review will be conducted in accordance with Joanna Briggs Institute (JBI) methodology for systematic reviews of effectiveness [34] and reported in accordance with the PRISMA guidelines [35]. PRISMA for systematic review protocol (PRISMA-P) was used to draft the protocol. The review is registered with PROSPERO (CRD42022322083). 

### 4.1. Search Strategy

The search strategy have been developed following a Population Intervention Comparator Outcome and Study Design (PICOS) framework. A combination of Medical Subject Headings (MESH) terms and keywords using Boolean operators, spelling variations, phrase searching, and truncation have been devised to increase sensitivity and ensure satisfactory search retrieval. Two reviewers (K.R. and N.C.), having experience with database searching, with consultation from a Health Sciences Librarian, have pre-test the search strategy in the Medline (OVID) (Table 1). Once the Medline search is finalised, the search will be subsequently adapted to the syntax and subject headings of the other databases. Finally, a final hand search of the reference lists of relevant studies that match inclusion criteria and previously published systematic reviews will be conducted to identify further eligible studies. 

The following electronic databases will be searched, without any restriction on publication date, type, language, or region: Medline (OVID), The Cochrane Library, Embase, Web of Science, Google Scholar and OT Seeker. For this review, articles will be included with no restriction on language as any relevant non-English articles will be attempted to be translated into English.

### 4.2. Study Selection

Studies identified by electronic databases and study citations from screening will be imported into EndNote X9 (Clarivate Analytics, Philadelphia, PA, USA), and duplicates removed. Following a pilot test, two independent reviewers (K.R. and A.A.) will screen the title and abstracts against the strict inclusion/exclusion criteria, and if unclear, the full text will be retrieved. Articles that meet the inclusion criteria will be retrieved in full and their details imported into the JBI System for the Unified Management, Assessment and Review of Information (JBI SUMARI) [36]. Two reviewers (K.R. and A.A.) will then independently assess the full-text articles and decide whether these meet the eligibility criteria. If required, the study authors will be contacted to seek additional information. Any disagreements that arise between the reviewers at any stage of the selection process will be resolved through discussion including a third reviewer (R.C.). If multiple reports are published from a single study, these will be linked together. Throughout this process, all reasons for exclusion of papers at the full text review stage will be recorded. The results of the study selection process will be presented in a PRISMA flow diagram [35].

### 4.3. Assessment of Methodological Quality

Two reviewers (K.R. and R.C.) will independently assess the methodological quality of each included study in this review. The Cochrane tool for assessing risk of bias in randomised trials (RoB-2 tool for cross-over trails) will be used [37]. Any disagreements that arise between the reviewers will be resolved through discussion and include a third reviewer (A.A.). Authors of papers will be contacted to request missing or additional data for clarification, where required. If a response is not received after two contact attempts, we will assess the study based on its available information. The level of risk of bias in each of these domains will be presented separately for each study in tables, figures and contextualised in a descriptive format in the final review publication. This narrative will outline the methodological issues and how these influenced the interpretation of the results. All studies included in this review will undergo data extraction and synthesis irrespective of the results of their methodological quality.

### 4.4. Data Extraction

A standardised data extraction form has been developed and will be pilot tested (on one study), and subsequently refined to ensure that we capture all relevant data (see Appendix A; Table A1, Table A2 and Table A3). A calibration exercise will be performed to ensure consistency across reviewers. Two review authors (K.R. and A.A.) will independently extract data, discrepancies will be identified and resolved through discussion with a third author (N.C.) where necessary. Authors of papers will be contacted to request missing or additional data, where required. If a response is not received after two contact attempts, we will assess the study based on its available information. The data extracted will be entered into an excel sheet including specific details about the study: article details, participant characteristics, intervention description, outcome measures and funding.

### 4.5. Data Synthesis

If possible, individual studies will be pooled for a statistical meta-analysis using STATA v15 (StataCorp, College Station, TX, USA) [38]. An assessment of the studies’ suitability for pooled analysis will be made following the data extraction process. Odds ratios (for dichotomous data) or weighted (or standardised) final post-intervention mean differences (for continuous data) will be used to calculate effect sizes, and their 95% confidence intervals will be calculated for analysis. The degree of statistical heterogeneity will be assessed using standard I-squared and Chi-squared statistics [39]. Some degree of heterogeneity is expected across the studies; therefore, the random effects model for meta-analysis will be applied [40]. Subgroup analyses will be conducted where there are sufficient data (if over 10 studies) based on age, diagnosis, and severity. Sensitivity analyses will be conducted to test the impact of risk of bias of included studies on outcomes. Where statistical pooling is not possible and/or there is substantial heterogeneity, a narrative synthesis of the study findings including tables and figures will be provided. Publication bias will be assessed using a funnel plot if there are 10 or more studies included in a meta-analysis [41]. Statistical tests for funnel plot asymmetry (Egger test, Begg test, Harbord test) will be performed where appropriate.

## 5. Conclusions

This systematic review aims to assess the effectiveness of SADE to reduce dental anxiety and corresponding negative behaviours and psychophysiology responses in children and young people with IDD. The findings from this review may widen the scope of occupational therapy to use an ecological and sensory lens in collaboration with dentists. This review may be essential to increase evidence-based practice and synthesise evidence available to influence greater oral health care outcomes for IDD population.

## Figures and Tables

**Table 1:** Medline (OVID) search strategy.

Search	Query	Records Retrieved *
#1	(Child* or adolescen* or teen or youth or young adult or pe?diatric* or preschool or infant*).ti,ab.	2,231,960
#2	Child, Preschool/ or Pediatrics/ or Adolescent/ or Child/ or infant/ or young adult/	4,006,547
#3	#1 or #2	4,667,012
#4	(Developmental disability* or intellectual disability* or special need* or mental retardation or disable* or autis* or ADHD or ASD or Cerebral palsy or attention deficit hyperactivity disorder or Down Syndrome or Fragile X Syndrome or Fetal alcohol spectrum disorder).ti,ab.	239,671
#5	Attention Deficit Disorder with Hyperactivity/ or Cerebral Palsy/ or Autistic Disorder/ or Disabled Persons/ or Disabled Children/ or child development disorders, pervasive/ or developmental disabilities/ or intellectual disability/ or Down Syndrome/ or Fragile X Syndrome/ or Fetal Alcohol Spectrum Disorders/	235,101
#6	#4 or #5	355,890
#7	#3 and #6	200,849
#8	((Dental adj3 (sensory adapted environment* or Multi-sensory environment*)) or Snoezelen or SADE).ti,ab.	199
#9	Health facility environment/ or environment/ or dental offices/ or environmental adaption/ or Environment, Controlled/	78,822
#10	#8 or #9	78,997
#11	(((Oral or dental) adj (health or hygiene or anxiety)) or behaviour or compliance or physiological or pain or arousal or stress or psychological).ti,ab.	2,606,469
#12	Stress, Psychological/ or Adaptation, Psychological/ or Psychological Distress/ or Arousal/ or Pain Perception/ or Pain/ or Dental Care/ or Dental Anxiety/ or Sensation/ or Patient Compliance/ or “Treatment Adherence and Compliance”/ or Oral Health/ or Oral hygiene/	512,327
#13	#11 or #12	2,832,243
#14	#7 and #10 and #13	161
NO LIMITS		

* Medline (OVID) search was conducted on 18 August 2022.

## Data Availability

Not applicable.

## Appendix A. Data Extraction Instrument

**Table A1:** Characteristics of included studies.

Article Details	Participant Characteristics	Dental Procedure Completed	SADE Adaptions	RDE Description	Outcomes Measures	Funding
Reference:	Sample size:					
Country:	Sex:					
Study design:	Age:					
	Diagnosis:					
	Demographic:					
	Other:					

**Table A2:** Description of the intervention.

Author	Visual	Tactile	Auditory	Additional Aspects/Comments
				
				

**Table A3:** Summary of the results of included studies.

Author	Data Analysis Undertaken	Findings (Raw Data)	Findings (Narrative/Summary)	Article Conclusions	Reviewers’ Conclusions	Limitations
						
						

## References

[1] American Psychiatric Association (2013). Diagnostic and Statistical Manual of Mental Disorders: DSM-5.

[2] Jin L.J., Lamster I.B., Greenspan J.S., Pitts N.B., Scully C., Warnakulasuriya S. (2016). Global burden of oral diseases: Emerging concepts, management and interplay with systemic health. Oral Dis..

[3] Anderson L.L., Larson S.A., MapelLentz S., Hall-Lande J. (2019). A Systematic Review of U.S. Studies on the Prevalence of Intellectual or Developmental Disabilities Since 2000. Intellect Dev. Disabil..

[4] Australian Institute of Health Welfare (2020). Australia’s Children.

[5] Emerson E. (2012). Deprivation, ethnicity and the prevalence of intellectual and developmental disabilities. J. Epidemiol. Community Health.

[6] Emerson E., Baines S. (2011). Health inequalities and people with learning disabilities in the UK. Tizard Learn. Disabil. Rev..

[7] Zhou N., Wong H.M., Wen Y.F., McGrath C. (2017). Oral health status of children and adolescents with intellectual disabilities: A systematic review and meta-analysis. Dev. Med. Child. Neurol..

[8] Ward L., Cooper S., Hughes-McCormack L., Macpherson L., Kinnear D. (2019). Oral health of adults with intellectual disabilities: A systematic review. J. Intellect. Disabil. Res..

[9] Song J.-S., Hyun H.-K., Shin T.J., Kim Y.-J. (2018). Effects of dental treatment and systemic disease on oral health-related quality of life in Korean pediatric patients. BMC Oral Health.

[10] Petrovic B.B., Peric T.O., Markovic D.L., Bajkin B.B., Petrovic D., Blagojevic D.B., Vujkov S. (2016). Unmet oral health needs among persons with intellectual disability. Res. Dev. Disabil..

[11] Wilson N.J., Lin Z., Villarosa A., George A. (2019). Oral health status and reported oral health problems in people with intellectual disability: A literature review. J. Intellect. Dev. Disabil..

[12] Byrappagari D., Jung Y., Chen K. (2018). Oral health care for patients with developmental disabilities: A survey of Michigan general dentists. Spec. Care Dent..

[13] Dahlander A., Soares F., Grindefjord M., Dahllöf G. (2019). Factors Associated with Dental Fear and Anxiety in Children Aged 7 to 9 Years. Dent. J..

[14] Lee J., Chang J. (2021). Oral health issues of young adults with severe intellectual and developmental disabilities and caregiver burdens: A qualitative study. BMC Oral Health.

[15] Armfield J.M. (2010). Towards a better understanding of dental anxiety and fear: Cognitions vs. experiences. Eur. J. Oral Sci..

[16] Klingberg G., Broberg A.G. (2007). Dental fear/anxiety and dental behaviour management problems in children and adolescents: A review of prevalence and concomitant psychological factors. Int. J. Paediatr. Dent..

[17] Morgan A.G., Rodd H.D., Porritt J.M., Baker S.R., Creswell C., Newton T., Williams C., Marshman Z. (2017). Children’s experiences of dental anxiety. Int. J. Paediatr. Dent..

[18] Keleş S., Abacıgil F., Adana F., Yeşilfidan D., Okyay P. (2018). The Association Between Dental Anxiety and Oral Health Related Quality of Life Among Individuals with Mild Intellectual Disability. Meandros Med. Dent. J..

[19] Shimizu V.T., Bueno O.F.A., Miranda M.C. (2014). Sensory processing abilities of children with ADHD. Braz. J. Phys. Ther..

[20] Lane A.E., Young R.L., Baker A.E.Z., Angley M.T. (2009). Sensory Processing Subtypes in Autism: Association with Adaptive Behavior. J. Autism. Dev. Disord..

[21] Dellapiazza F., Michelon C., Oreve M.-J., Robel L., Schoenberger M., Chatel C., Vesperini S., Maffre T., Schmidt R., Blanc N. (2019). The Impact of Atypical Sensory Processing on Adaptive Functioning and Maladaptive Behaviors in Autism Spectrum Disorder During Childhood: Results From the ELENA Cohort. J. Autism. Dev. Disord..

[22] Chadwick D., Chapman M., Davies G. (2018). Factors affecting access to daily oral and dental care among adults with intellectual disabilities. J. Appl. Res. Intellect. Disabil..

[23] Mac Giolla Phadraig C., Asimakopoulou K., Daly B., Fleischmann I., Nunn J. (2020). Nonpharmacological techniques to support patients with intellectual developmental disorders to receive dental treatment: A systematic review of behavior change techniques. Spec. Care Dent..

[24] LeBel J., Nunno M.A., Mohr W.K., O’Halloran R. (2012). Restraint and Seclusion Use in U.S. School Settings: Recommendations From Allied Treatment Disciplines. Am. J. Orthopsychiatry.

[25] Deshais M.A., Fisher A.B., Hausman N.L., Kahng S. (2015). Further investigation of a rapid restraint analysis. J. Appl. Behav. Anal..

[26] Martins-Junior P.A. (2017). Dental treatment under general anaesthetic and children’s oral health-related quality of life: Question: What is the impact of dental treatment under general anaesthesia on children’s oral health-related quality of life?. Evid.-Based Dent..

[27] Potter C.N., Wetzel J.L., Learman K.E. (2019). Effect of sensory adaptations for routine dental care in individuals with intellectual and developmental disabilities: A preliminary study. J. Intellect. Dev. Dis..

[28] Kim G., Carrico C., Ivey C., Wunsch P.B. (2019). Impact of sensory adapted dental environment on children with developmental disabilities. Spec. Care Dent..

[29] Sigal A., Sigal M. (2022). The Multisensory/Snoezelen Environment to Optimize the Dental Care Patient Experience. Dental Clin..

[30] Bodison S.C., Diane P.L. (2018). Specific Sensory Techniques and Sensory Environmental Modifications for Children and Youth With Sensory Integration Difficulties: A Systematic Review. Am. J. Occup. Ther..

[31] Ismail A., Tengku Azmi T., Malek W., Mallineni S. (2021). The effect of multisensory-adapted dental environment on children’s behavior toward dental treatment: A systematic review. J. Indian Soc. Pedod. Prev. Dent..

[32] World Health Organization (2002). Towards a Common Language for Functioning, Disability, and Health: ICF.

[33] Faulks D., Norderyd J., Molina G., Phadraig C.M., Scagnet G., Eschevins C., Hennequin M. (2013). Using the International Classification of Functioning, Disability and Health (ICF) to Describe Children Referred to Special Care or Paediatric Dental Services. PLoS ONE.

[34] Tufanaru C., Munn Z., Aromataris E., Campbell J., Hopp L., Aromataris E., Munn Z. (2020). Chapter 3: Systematic reviews of effectiveness. JBI Manual for Evidence Synthesis.

[35] Page M.J., McKenzie J.E., Bossuyt P.M., Boutron I., Hoffmann T.C., Mulrow C.D., Shamseer L., Tetzlaff J.M., Akl E.A., Brennan S.E. (2021). The PRISMA 2020 statement: An updated guideline for reporting systematic reviews. BMJ.

[36] Munn Z., Aromataris E., Tufanaru C., Stern C., Porritt K., Farrow J., Lockwood C., Stephenson M., Moola S., Lizarondo L. (2019). The development of software to support multiple systematic review types: The Joanna Briggs Institute System for the Unified Management, Assessment and Review of Information (JBI SUMARI). Int. J. Evid. Based Healthc..

[37] Sterne J.A., Savović J., Page M.J., Elbers R.G., Blencowe N.S., Boutron I., Cates C.J., Cheng H.-Y., Corbett M.S., Eldridge S.M. (2019). RoB 2: A revised tool for assessing risk of bias in randomised trials. BMJ.

[38] Valentine J.C., Pigott T.D., Rothstein H.R. (2010). How many studies do you need? A primer on statistical power for meta-analysis. J. Educ. Behav. Stat..

[39] Higgins J.P., Thomas J., Chandler J., Cumpston M., Li T., Page M.J., Welch V.A. (2019). Cochrane Handbook for Systematic Reviews of Interventions.

[40] Tufanaru C., Munn Z., Stephenson M., Aromataris E. (2015). Fixed or random effects meta-analysis? Common methodological issues in systematic reviews of effectiveness. Int. J. Evid.-Based Healthc..

[41] Sterne J.A., Sutton A.J., Ioannidis J.P., Terrin N., Jones D.R., Lau J., Carpenter J., Rücker G., Harbord R.M., Schmid C.H. (2011). Recommendations for examining and interpreting funnel plot asymmetry in meta-analyses of randomised controlled trials. BMJ.

